# Genetic patterns in peripheral marine populations of the fusilier fish *Caesio cuning* within the Kuroshio Current

**DOI:** 10.1002/ece3.4644

**Published:** 2018-11-14

**Authors:** Amanda S. Ackiss, Christopher E. Bird, Yuichi Akita, Mudjekeewis D. Santos, Katsunori Tachihara, Kent E. Carpenter

**Affiliations:** ^1^ Department of Biological Sciences Old Dominion University Norfolk Virginia; ^2^ Department of Life Sciences Texas A&M University – Corpus Christi Corpus Christi Texas; ^3^ Okinawa Prefectural Fisheries Research and Extension Center Itoman Okinawa Japan; ^4^ Genetic Fingerprinting Laboratory National Fisheries Research and Development Institute Quezon City Philippines; ^5^ Laboratory of Fisheries Biology & Coral Reef Studies, Faculty of Science University of the Ryukyus Ryukyus Okinawa Japan

**Keywords:** dispersal, gene flow, genetic divergence, genetic diversity, peripheral populations, RAD sequencing

## Abstract

**Aim:**

Mayr's central‐peripheral population model (CCPM) describes the marked differences between central and peripheral populations in genetic diversity, gene flow, and census size. When isolation leads to genetic divergence, these peripheral populations have high evolutionary value and can influence biogeographic patterns. In tropical marine species with pelagic larvae, powerful western‐boundary currents have great potential to shape the genetic characteristics of peripheral populations at latitudinal extremes. We tested for the genetic patterns expected by the CCPM in peripheral populations that are located within the Kuroshio Current for the Indo‐Pacific reef fish, *Caesio cuning*.

**Methods:**

We used a panel of 2,677 SNPs generated from restriction site‐associated DNA (RAD) sequencing to investigate genetic diversity, relatedness, effective population size, and spatial patterns of population connectivity from central to peripheral populations of *C. cuning* along the Kuroshio Current.

**Results:**

Principal component and cluster analyses indicated a genetically distinct lineage at the periphery of the *C. cuning* species range and examination of SNPs putatively under divergent selection suggested potential for local adaptation in this region. We found signatures of isolation‐by‐distance and significant genetic differences between nearly all sites. Sites closest to the periphery exhibited increased within‐population relatedness and decreased effective population size.

**Main Conclusions:**

Despite the potential for homogenizing gene flow along the Kuroshio Current, peripheral populations in *C. cuning* conform to the predictions of the CCPM. While oceanography, habitat availability, and dispersal ability are all likely to shape the patterns found in *C. cuning* across this central‐peripheral junction, the impacts of genetic drift and natural selection in increasing smaller peripheral populations appear to be probable influences on the lineage divergence found in the Ryukyu Islands.

## 1 INTRODUCTION

Within the spatial distribution of a species, peripheral populations can be prone to edge effects that significantly alter their genetic characteristics relative to central counterparts. This phenomenon is summarized in the central‐peripheral population model (CPPM; Mayr, 1963). Under the CPPM, populations at the center of a species range are contiguous, abundance is regulated by density‐dependent factors, and gene flow is multidirectional. These combined forces result in larger central populations with high genetic diversity. In contrast, peripheral populations are often fragmented and gene flow occurs from a single direction. Isolation, coupled with higher selective pressure in marginal habitats, results in reduced population sizes, low genetic diversity, and genetic divergence (Gould, 2002; Nei, Maruyama, & Chakaborty, 1975).

Empirical research indicates that the majority of species approximately conform to the CPPM but studies of marine taxa lag behind those of terrestrial taxa (Ecker, Samis, & Lougheed, 2008). Most marine organisms disperse via a larval pelagic phase, and a large range of dispersal potential exists among marine species due to differences in duration of the pelagic period, ability to orient, and oceanographic patterns complicating the predictability of population connectivity (Cowen, Gawarkiewicz, Pineda, Thorrold, & Werner, 2007; Kinlan, Gaines, & Lester, 2005; Selkoe, Gaggiotti, Bowen, & Toonen, 2014; Shanks, 2009; Weersing & Toonen, 2009). Scientists consider species with disjunct peripheral populations and low genetic diversity to have higher evolutionary value than those with continuous peripheral populations (Bunnell, Campbell, & Squires, 2004; Lesica & Allendorf, 1995). Therefore, it is important to increase our understanding of marine species and geographic regions prone to edge effects.

One region of interest for marine species is the Ryukyu Islands, which mark the northern periphery of many ubiquitous coral reef species’ ranges in the western Pacific Ocean (Randall, 1998; Veron & Minchin, 1992). This trend is generally attributed to the steady decrease in sea surface temperature to beyond the thermodynamic threshold of tropical reef organisms at increasingly higher latitudes (Crossland, 1984; Dana, 1843; Jokiel & Coles, 1977; Rosen, 1975; Vaughan, 1919). Hermatypic corals are obligatory habitat for many tropical reef species and their prey, and at the latitude of the southernmost main island of Japan, shallow water reefs are classified as marginal due to low surface temperature, low aragonite saturation states, and low light (Kleypas, McManus, & Meñez, 1999). Without supportive habitat and beyond the extent of their fundamental niche, most tropical reef organisms do not succeed in establishing populations above the Ryukyus, though range expansions are beginning to be documented with rising global sea surface temperatures (Yamano, Sugihara, & Nomura, 2011).

The steady, northerly stream of warm surface water flowing through the Ryukyu Islands that results in the persistence of coral reef organisms at higher‐than‐normal latitudes is provided by a powerful western‐boundary current known as the Kuroshio Current (Veron, 1992; Yamano, Hori, Yamauchi, Yamagawa, & Ohmura, 2001), but the role that western boundary currents such as the Kuroshio Current and its Atlantic Ocean counterpart, the Gulf Stream, may play in shaping the genetic patterns in the northern peripheral populations of tropical species relative to central counterparts remains mostly unexamined. The only taxon sampled across the flow path of the Kuroshio Current to address questions about genetic connectivity and diversity in central and peripheral populations is passive propagule‐dispersing seagrass (Arriesgado et al., 2015, 2016 ; Kurokochi et al., 2016; Nakajima et al., 2014) leaving a majority of the tropical species and ecosystems whose peripheral populations are found in the Ryukyu Islands unstudied.

**Figure 1:**
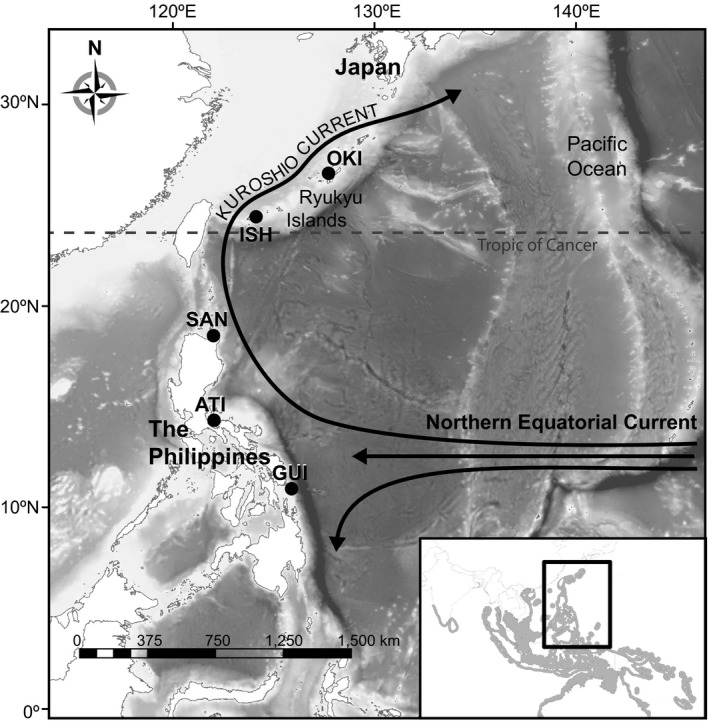
*Caesio cuning* collection sites along the Kuroshio Current. Okinawa (OKI), Ishigaki (ISH), Santa Ana (SAN), Atimonan (ATI), Guiuan (GUI). Dashed line indicates the Tropic of Cancer, the line of latitude widely considered the northern limit to tropical systems (23.5°N). INSET: The sampling area (box) within the *C. cuning* species range (gray shading)

The pathway of the Kuroshio Current is an intriguing setting for examining central‐peripheral patterns in shallow water tropical marine populations since it contains both the topography to support disjunct populations and the oceanography to support continuous populations. The Kuroshio is formed when the Northern Equatorial Current (NEC) hits and bifurcates north and south at ~13°N along the eastern coastline of the Philippines (Figure 1; Nitani, 1972; Toole, Millard, Wang, & Pu, 1990). It flows past nearly 1,000 km of contiguous shoreline habitat and crosses a large expanse of deep water to the disjunct, sub‐tropical island reef systems that make up the Ryukyu Islands before finally turning east off the coast of Honshu, Japan to form the North Pacific Current. However, it can take as little as one effective migrant per generation to nullify disruptive genetic drift between two populations (Spieth, 1974), and since the Kuroshio Current can reach mean maximum surface velocities of ~1.2 m/s (~104 km/day) (Yang et al., 2015) there is great potential for genetic continuity via the dispersive larvae characteristic of most marine organisms.

As one of the many tropical species whose northern distribution ends in the Ryukyu Islands, the Redbelly Yellowtail Fusilier, *Caesio cuning* (Bloch, 1791), has the potential for either disjunct or continuous peripheral populations within the Kuroshio Current. Like the majority of reef organisms, *C. cuning* has a bipartite life history beginning as pelagic larvae and settling on coral reefs as juveniles. In contrast to other fusiliers, such as those in the genera *Pterocaesio* and *Dipterygonotus*, it has been noted that *C. cuning* larvae are primarily “coastal”, being nearly always found over the mid to inner continental shelf (Reader & Leis, 1996). Observations of eleven *C. cuning* larvae in situ also showed some evidence that larvae can detect and orient themselves toward reefs (Leis & Carson‐Ewart, 2003). Adults are non‐migratory and dependent on reef structure for protection at night. These observations suggest that long distance dispersal in *C. cuning* is unlikely without the presence of a strong oceanographic conduit such as the Kuroshio Current. And as an exploited fishery throughout its range, a better understanding of whether *C. cuning* peripheral populations are continuous or disjunct will be useful for effective management.

Here, restriction site‐associated DNA (RAD) sequencing (ezRAD; Toonen et al., 2013) was used to examine fine‐scale genetic signatures in *C. cuning* collected from the center of the species range in the Philippines to the northern edge of the species range in the Kuroshio Current. A panel of 2,677 neutral single nucleotide polymorphisms (SNPs) was used to address to two main questions: (a) do peripheral *C. cuning* populations within this western boundary current exhibit continuous or disjunct populations, and (b) are the genetic signatures of edge effects present that are expected by the CPPM from central to peripheral populations?

## 2 MATERIALS AND METHODS

Pectoral fin and muscle tissue were collected from 307 fish at regional markets and landings from three sites below the latitude which marks the standard tropical‐subtropical boundary (23.5°N) along the east coast of the Philippines and two sites at the northern edge of the species range above 23.5°N in the Ryukyu Islands of Japan (Figure 1, Table 3). Tissues were stored in 95% ETOH or DNA/RNA Shield™ (Zymo Research), and DNA was extracted using E‐Z 96^®^ Tissue DNA Kits (Omega Bio‐tek, Inc.). Elutions containing high‐weight DNA were quantified with a fluorescence microplate reader, and aliquots containing 100 ng of DNA were cleaned using AMPureXP beads (Beckman Coulter, Inc.) for input into library preparation.

### 2.1 RAD library preparation and sequencing

Restriction site‐associated DNA (RAD) libraries were prepared following a modified ezRAD protocol (Toonen et al., 2013). Genomic DNA was cut at 5’‐GATC sites using the isoschizomers *MboI* and *Sau3AI* (New England Biolabs). Cleaned template DNA bound by AMPureXP beads was eluted in 21.5 µl of water and digested overnight with 2.5 uL of CutSmart^®^ Buffer and 2.5 U of each restriction enzyme in 25 µl reactions. Digested DNA was rebound to beads using a 2× 3 M NaCl, 20% PEG solution (Faircloth & Glenn, 2014; Fisher et al., 2011) and cleaned before being input into an Illumina TruSeq Nano DNA HT Library Prep Kit at the end repair step. DNA was size‐selected for 350‐bp fragments and dual‐indexed adaptors ligated. Libraries were enriched with an 8‐cycle PCR reaction using manufacturer's cycling parameters. Ligation was confirmed and libraries quantified using qPCR amplification with a KAPA Library Quant Kit (KAPA Biosystems). The library products were run through a BluePippin (Sage Science) using 2% agarose dye‐free cassettes for additional size‐selection, which was confirmed via a Fragment Analyzer™ (Advanced Analytical Technologies), and reduced libraries for 205 individuals were paired‐end sequenced (PE 100) on an Illumina HiSeq 2,500 and 4,000 at 60 libraries per lane.

### 2.2 Read processing, SNP filtering, and outlier detection

Forward and reverse reads were truncated to 90 bp and quality filtered using the Stacks subprogram process_radtags (Catchen, Hohenlohe, Bassham, Amores, & Cresko, 2013) before being input into the dDocent pipeline (Puritz, Hollenbeck, & Gold, 2014) for de novo assembly, read mapping, and variant calling. Default dDocent parameters were modified based on performance, including increasing the CD‐HIT sequence similarity parameter (‐c) to 0.92 and increasing the minimum base phred score (‐q) to 20 for an allele to be included in variant calling with Freebayes v1.0.2–58‐g054b257 in order to decrease the overall number of RAD contigs that had more than two haplotypes present after mapping.

Filtering parameters were modified from recommendations in dDocent content ( https://github.com/jpuritz/dDocent/). Preliminary filtering to remove samples with high amounts of missing data and erroneous calls was done with VCFtools (Danecek et al., 2011). Conditions under which variants were removed were as follows: quality value lower than 30, genotyped in fewer than 95% of individuals, a minor allele frequency less than 0.05, a mean coverage depth less than five, and sites with more than two alleles. Additional filtering with the program vcflib ( https://github.com/ekg/vcflib) removed loci with a heterozygote allele balance <0.25 and>0.75, reads from both strands, large variation in mapping quality among alleles, an alternate allele only supported by unpaired reads, and a mean depth of well above the majority distribution of depths across loci since high coverage can lead to inflated quality scores or false heterozygotes (Li, 2014). Remaining variants were decomposed to remove indels.

Genetic structure between populations will generate significant departures from Hardy‐Weinberg Equilibrium (HWE) in a global dataset (Wahlund, 1928), so SNPs were filtered for population‐specific deviation from HWE. Samples with more than 10% missing data after primary filtering were removed, as well as samples or loci with high heterozygosity (>0.6). Multiple SNPs on a single RAD tag are linked by proximity, so one random SNP per paired‐end tag was selected for the final panel. Dataset file format conversions were executed with the Java conversion tool PGDSpider v 2.0.8.3 (Lischer & Excoffier, 2012).

The filtered panel of SNPs was tested for outliers using both frequentist and Bayesian approaches with the programs LOSITAN (Antao, Lopes, Lopes., Beja‐Pereira, & Luikart, 2008; Beaumont & Nichols, 1996) and BayeScan v2.1 (Foll & Gaggiotti, 2008), respectively. LOSITAN employs an *F*_ST_‐outlier approach for selection detection, evaluating the relationship between *F*_ST_ and *H*_e_. LOSITAN was run using the infinite alleles mutation model with parameter settings of “neutral” and forced mean *F*_ST_ (recommended), 500,000 simulations, confidence interval of 0.95, and a subsample size of 40. BayeScan uses a Bayesian approach to selection detection that implements the multinomial‐Dirichlet model and was run with default parameters. A false discovery rate (FDR) correction (α = 0.05) was set in both programs, and any loci identified as outliers by either program after correction were removed to produce a neutral panel of SNPs for our analyses. Both forward and reverse tags associated with outlier loci were run through the megablast search tool in BLASTN v2.6.1 to examine similarity to NCBI archived sequences in the nr/nt and wgs databases (Morgulis et al., 2008; Zhang, Schwartz, Wagner, & Miller, 2000).

### 2.3 Genetic differentiation and spatial clustering

Multiple approaches were used to examine the level of genetic connectivity among sites. Pairwise genetic differentiation between populations (*F*_ST_) was generated from putatively neutral SNPs in the program GenoDive v2.0b27 (Meirmans & Van Tienderen, 2004). Significance of *F*_ST_ values was tested using 10,000 permutations, and p‐values were corrected using Benjamini & Hochberg's method of FDR correction (Benjamini & Hochberg, 1995). Genetic variability within and among sites was examined in both neutral and putatively adaptive loci with a principal components analysis (PCA) in the R package “adegenet” (Jombart, 2008; Jombart & Ahmed, 2011). All remaining analyses were run exclusively on the panel of neutral loci.

The power of different methods to estimate the number of discrete genetic populations within a dataset can vary based on a species’ dispersal characteristics and the spatial distribution of sampling (Murphy, Evans, Cushman, & Storfer, 2008; Schwartz & McKelvey, 2008), therefore the use of multiple methods to detect barriers is recommended (Blair et al., 2012). The program STRUCTURE v2.3.4 (Falush, Stephens, & Pritchard, 2003; Pritchard, Stephens, & Donnelly, 2000) was used to identify distinct genetic clusters and assign individuals to populations under the assumption of population admixture and correlated allele frequency. Runs consisted of a burn‐in period of 50,000 MCMC iterations followed by 100,000 iterations for inferred K of 1 to 5 and replicated 10 times for consistency. Population identity was not used as an a priori parameter for clustering. Results were collated in STRUCTURE HARVESTER (Earl & vonHoldt, 2012), and the log probability and ΔK statistic (Evanno, Regnaut, & Goudet, 2005) were used to determine the most likely number of clusters. Replicate Q‐matrices for the optimal K were processed with the Greedy algorithm in CLUMPP v1.1.2 (Jakobsson & Rosenberg, 2007) in order to produce a single optimal alignment. Individuals were assigned to the cluster with the largest percentage of membership. For comparison, the spatially explicit clustering program GENELAND (Guillot, 2008; Guillot & Santos, 2009; Guillot, Estoup, Mortier, & Cosson, 2005; Guillot, Mortier, & Estoup, 2005) was used to approximate the pattern of population spread across space. Ten replicates of the spatial model in GENELAND v4.0.8 were run through R command line (R Core Team, 2015) with the correlated allele frequency model using recommended starting parameters: number of possible clusters (K) set to vary between 1 and 10 with 100,000 Markov Chain Monte Carlo (MCMC) iterations, thinning of 100, burn‐in of 200, maximum rate of the Poisson process fixed to 174, and maximum number of nuclei in the Poisson‐Voronoi tessellation process fixed to 348. Samples from each location shared the same spatial coordinates so to allow individuals with the same coordinates to be assigned to different populations, UTM coordinate uncertainty was set to 1 km. The modal K from these initial runs was fixed for 20 additional replicates, and the run with the highest posterior probability was used to assign individuals to clusters.

Distinguishing spatial clustering from clinal genetic differentiation can be challenging since tests for one can be biased by the presence of the other (Guillot, Leblois, Coulon, & Frantz, 2009; Meirmans, 2012; Schwartz & McKelvey, 2008). One suggested method of distinguishing a pattern of isolation‐by‐distance (IBD) from spatial clustering is running partial Mantel tests. Shortest overwater distances between collection sites were calculated in ArcGIS v10.1 using the Cylindrical Equal Area projected coordinate system and a 500 m cell size (2,500 m^2^). Distances were estimated using the cost distance tool, assigning an equal “cost” to each cell of water and eliminating all paths that crossed land. Correlation of geographic distances to pairwise *F*_ST_ was examined with a basic Mantel test. A third matrix of cluster membership by genetic clusters identified in previous analyses was used for partial Mantel tests. Mantel and partial Mantel tests were run in the R package “vegan” (Oksanen et al., [Link]) and the significance of Mantel statistics was measured with 120 permutations of the matrices.

### 2.4 Genetic diversity, effective population size, and relatedness

To examine sites for the presence of edge effects, we assessed genetic diversity, effective population size, and relatedness within sampled sites. Measures of genetic diversity were generated in Genodive. Estimates of contemporary effective population (*N*_e_) size were calculated using a bias‐corrected linkage disequilibrium (LD) method in the program NeEstimator v2.1 (Do et al., 2014). In addition to neutrality, the LD *N*_e_ method assumes independence of loci, so to test the effects of possible linkage on the *N*_e_ estimates, *N*_e_ was calculated with and without pairwise comparisons of r^2^ ≤ 0.5 using custom R scripts (Gruenthal et al., 2014; original scripts from Candy et al., 2015; modified script for parsing the LD Burrows coefficients output file from NeEstimator v2.1 provided here as supplemental material). Coefficients of relatedness (*r*) for individuals at each site were estimated using a moment estimator and two maximum‐likelihood estimators in the R package “related” (Pew, Muir, Wang, & Frasier, 2015), which have been shown to be robust methods for examining relatedness (Attard, Beheregaray, & Möller, 2018; Wang, 2011, 2014). One pair of individuals, San_007 and San_008, had a *r* value of 0.97 which is consistent with the same individual being sampled twice so San_008 was removed from all further analyses (Supporting Information, Table S1).

## 3 RESULTS

A total of 2,713 SNPs were retained after filtering. Outlier tests detected 36 loci putatively under divergent selection (11 of which were identified by both LOSITAN and BayeScan). A blast search of these 36 loci resulted in significant alignments for approximately 14% (5 RAD tags) to known genomic regions in other fishes, including *Dicentrarchus labrax*,* Oplegnathus fasciatus*,* Solea senegalensis*,* Stegastes partitus*, and *Larimichthys crocea*. One tag had a 94% identity match within the CYTH3 gene, and the rest produced 83%–100% identity matches to genomic DNA regions as close as 2,149 bp from known or predicted coding regions. A summary of matches can be found in Table S2 in Supporting Information. These 36 outlier loci were removed, and all analyses were performed on a neutral panel of 2,677 SNPs with the exception of the principal component analysis, which was run with both panels.

### 3.1 Genetic differentiation and spatial clustering

Genetic structure was evident among all locations, with the exception of the two most northern sites in the Philippines, Atimonan, and Santa Ana (*F*_ST_ = 0.0004–0.0132, Table 1). The greatest pairwise genetic differentiation was measured between the two most distant sites, Guiuan and Okinawa.

**Table 1:** Pairwise genetic and geographic distance values

Site	Okinawa (OKI)	Ishigaki (ISH)	Santa Ana (SAN)	Atimonan (ATI)	Guiuan (GUI)
OKI	–	504	1,080	1570	1718
ISH	0.0106**	–	694	1,184	1,495
SAN	0.0114**	0.0026**	–	541	997
ATI	0.0122**	0.0041**	0.0004	–	687
GUI	0.0132**	0.0046**	0.0008*	0.0009*	–

Notes

Pairwise *F*_ST_ values are reported in the lower diagonal, and pairwise geographic distances (in kilometers) are reported in the upper diagonal.

a Significant, *p* ≤ 0.0355.

b Significant, *p* = 0.0001.

**Figure 2:**
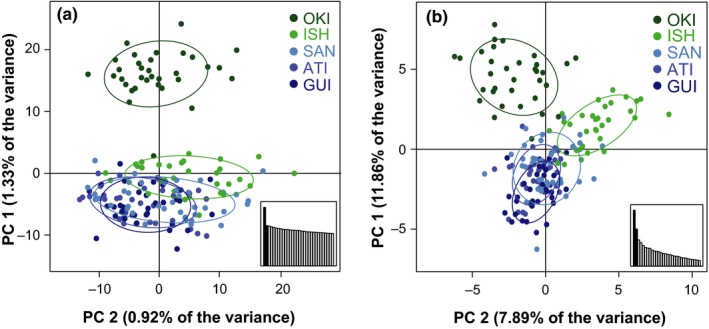
Principal component analysis of *C. cuning* along the Kuroshio Current. The first principal component (PC 1) is graphed along the y‐axis, and the second principal component (PC 2) is graphed along the x‐axis. Insets contain the first 30 eigenvalues. a: 174 individuals at 2,677 neutral SNPs. b: 174 individuals at 36 SNPs identified as being under positive selection

Principal component analysis (PCA) also indicated that the peripheral population in Okinawa was genetically different from the others. A PCA using the neutral SNP panel separated all but one of the Okinawa fish from those at other locations along the axis of the first principal component (Figure 2a). The first principal component (PC) accounted for only 1.33% of the total variance measured in the neutral panel of SNPs, but this is more than twice the variation of the average axis. A PCA performed on the 36 SNPs identified as outliers was similar to the PCA of neutral loci, but the fish from Ishigaki were also distinct, resulting in three clusters—one comprised of the samples from Okinawa, one comprised of the samples from Ishigaki, and one comprised of the Philippine sites (Figure 2b). PC one separates the two Ryukyu Island sites from the Philippines sites and accounts for 11.9% of the variance in these SNPs. No signal of lane or sequencer effects was found in any PCs.

**Figure 3:**
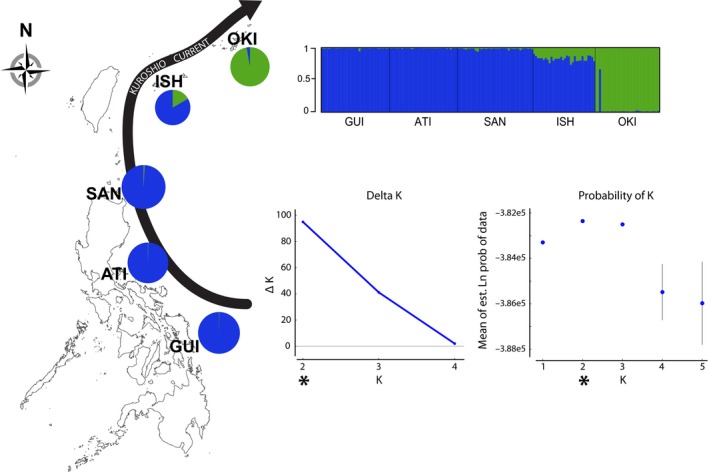
Results from a STRUCTURE analysis of central‐peripheral populations of *C. cuning*. K = 2 generated the largest ΔK (first graph) and log probability values (second graph) denoted by *. The STRUCTURE plot indicates the probability of membership to either lineage for each individual by location

STRUCTURE results were consistent with those from the PCAs, with all but one fish from the peripheral population of Okinawa belonging to an exclusive genetic cluster (Figure 3). Both the log‐likelihood [L(*K*)] and Δ*K* indicated that *K* = 2. The remaining Okinawan fish exhibited a profile similar to those from Ishigaki, which were assigned to a second cluster with all individuals from the central Philippines sites (Figure 3). The GENELAND run with the largest log‐likelihood score assigned the individuals from the three Philippine sites to one cluster, the individuals from Ishigaki to a second cluster, and the individuals from Okinawa to a third cluster.

**Figure 4:**
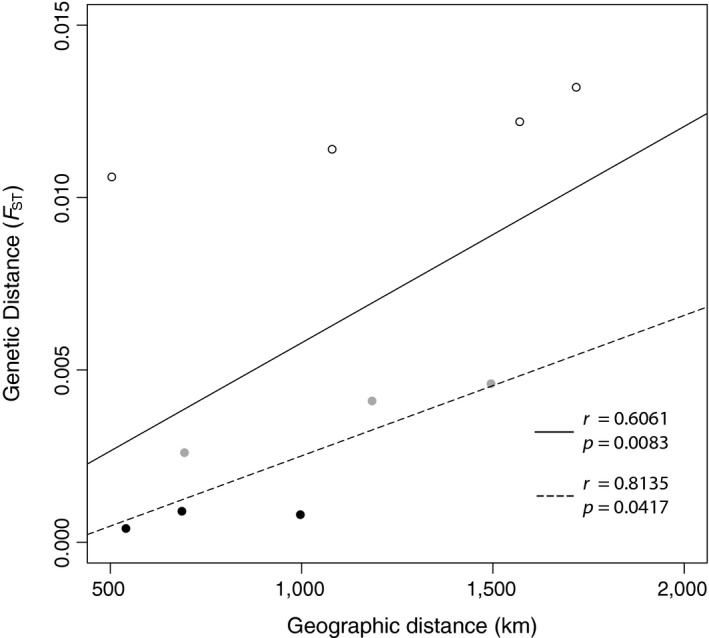
Isolation‐by‐distance (IBD) in *C. cuning* along the Kuroshio Current. Open circles: Okinawa versus all other sites, gray circles: Ishigaki versus Philippine sites, black circles: comparisons between Philippine sites. Regression lines are drawn from either all data (solid line) or data without Okinawa (dashed line) with corresponding Mantel statistics (r) and p‐values for each dataset in the bottom right.

A basic Mantel test was significant for correlation between genetic and geographic distance (*r* = 0.6061, *p* = 0.0083, Figure 4). The concurrent PCA and STRUCTURE results indicating the presence of hierarchical structure were used to inform cluster assignment for partial Mantels. Pairwise *F*_ST_ values between Okinawa and all other sites were assigned as one (different clusters) and all other pairwise *F*_ST_ values assigned as 0 (same cluster). The partial Mantel test for cluster‐corrected IDB resulted in a lower Mantel statistic than the test for IBD‐corrected clustering (*r* = 0.7894 and *r* = 0.9778, Table 2), but both were significant, indicating that clinal genetic differentiation and distinct hierarchical clustering are likely both contributing to structure observed between sites.

**Table 2:** Mantel and partial Mantel tests for isolation by distance (IBD)

	Test	*x*	*y*	*z*	*r*	*p*‐value
Mantel	IBD	*F*_ST_	Geographic distance	–	0.60610	0.0083
Partial Mantel	IBD	*F*_ST_	Geographic distance	Clusters	0.7894	0.0333
	Clusters	*F*_ST_	Clusters	Geographic distance	0.9778	0.0083

A matrix of cluster similarity was used for partial Mantel tests. Cluster assignment was informed by PCA and STRUCTURE results with Okinawa assigned to one cluster and all other sites to another. Significance of the Mantel statistic (*r*) was estimated with the maximum possible number of permutations (*n* = 120).

### 3.2 Genetic diversity, effective population size and relatedness

Summary statistics by site are reported in Table 3. Observed and expected heterozygosity (*H*_o_ */H*_e_) were similar across all sites, with site‐specific *H*_o_ ranging from 0.2543 in Guiuan (central) to 0.2617 in Okinawa (peripheral). The percentage of polymorphic loci observed in each site was greater than 99% indicating negligible levels of fixation in all sites for any loci in the final SNP panel.

**Table 3:** Site statistics for *Caesio cuning* along the Kuroshio Current

Location	abbv	GPS coordinates	Collection date	*n*	N	*A*_o_ (%)	*H*_o_	*H*_e_	*G*_IS_
Okinawa	OKI	26.47754 N, 127.80504 E	Sept 2014	56	33	99.1	0.2617	0.2587	−0.0119
Ishigaki	ISH	24.37328 N, 124.08956 E	April 2014	74	32	99.6	0.2594	0.2599	0.0019
Santa Ana	SAN	18.53085 N, 122.09347 E	April 2015	51	39	99.9	0.2603	0.2602	−0.0005
Atimonan	ATI	14.00466 N, 121.92334 E	May 2014	59	35	99.9	0.2615	0.2612	−0.0015
Guiuan	GUI	10.93783 N, 125.85169 E	June 2013	67	35	99.7	0.2543	0.2563	0.0080

Site code (abbv), number of tissues collected (*n*), individuals successfully genotyped and used in most analyses (*N*), percentage of observed alleles represented within site (*A*_o_), observed heterozygosity (*H*_o_), expected heterozygosity (*H*_e_), and the inbreeding coefficient (*G*_IS_, an analogue to *F*_IS_ based on *G*_ST_: Nei 1973).

Estimates of effective population size (*N*_e_) within sites ranged from 2,258–5,731 in Okinawa (peripheral) to “infinite” in Atimonan and Guiuan (central, Table 4). An “infinite” estimate is often an indication that the mean sample size (*S*) is too small to generate an estimate of *N*_e_ using the LD *N*_e_ method (Do et al., 2014; Waples & Do, 2010). When all pairwise locus comparisons were included in estimates (*r* ^2^ < 1), the smallest *N*_e_ was measured in Okinawa, with increasing estimates of *N*_e_ in Ishigaki and Santa Ana. Given that sample sizes were similar among all sites (*S* = 30.4–38.5), and these were adequate to generate finite estimates of *N*_e_ in Okinawa, Ishigaki, and Santa Ana but not Atimonan and Guiuan, it indicates that the true *N*_e_ in these two southern sites is larger than the true *N*_e_ of the northern sites, which conforms to our expectations. Exclusion of pairwise locus comparisons using a cutoff of *r* ^2^ ≤ 0.5 did affect estimates of *N*_e_, indicating the presence of some linkage between loci.

**Table 4:** *N*_e_ for *C. cuning* along the Kuroshio Current. *S* is the harmonic mean sample size for each population

Site	*S*	*N*_e_ (CI)	
		*r* ^2^ < 1	*r* ^2^ ≤ 0.5
OKI	31.4	2,258 (1,590–3,881)	5,731 (2,780‐Inf)
ISH	30.4	4,423 (2,422–25,010)	Inf (14,535‐Inf)
SAN	38.5	5,967 (3,317–29,345)	14,322 (4,941‐Inf)
ATI	33.2	Inf (6,509‐Inf)	Inf (Inf‐Inf)
GUI	34.5	Inf (33,833‐Inf)	Inf (Inf‐Inf)
ATI‐SAN	71.7	Inf (15,713‐Inf)	Inf (16,775‐Inf)
GUI‐ATI‐SAN	106.2	Inf (23,325‐Inf)	Inf (25,688‐Inf)
GUI‐ATI‐SAN‐ISH	136.7	34,814 (15,535‐Inf)	41,608 (17,107‐Inf)

Effective population size estimates (*N*_e_) and confidence intervals (CI) were generated in R using the *r* ^2^ values from the linkage disequilibrium method in NeEstimator with a minor allele frequency cutoff of 0.05. *N*_e_ for each location or combination of locations was calculated with all pairwise comparisons (*r* ^2^ < 1) and using only comparisons with *r* ^2^ ≤ 0.5.

**Figure 5:**
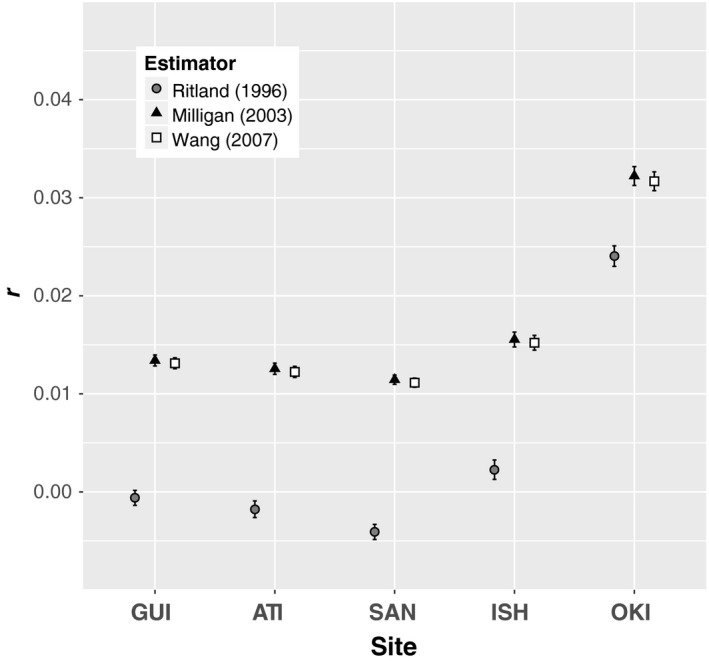
Relatedness among *C. cuning* within sites. Mean coefficients of relatedness (r) generated with three different estimators in the R package “related.” Standard error of the mean is denoted by bars

The peripheral population of Okinawa had the most genetically related fish, followed by the adjacent sampling location in Ishigaki (Figure 5). Pairwise coefficients of relatedness (*r*) across all individuals ranged from −0.085 to 0.167. Most pairwise comparisons (99.9%) were deemed unrelated. The relatedness of one pair of individuals from Ishigaki (*r* = 0.167, both maximum‐likelihood estimators, and *r* = 0.150, Ritland, 1996) and one pair of individuals from Okinawa *r* = 0.159, Milligan, 2003, *r* = 0.158, Wang, 2007, and *r* = 0.156, Ritland, 1996) was approximately that expected from first cousins. An additional 11 Okinawa‐Okinawa pairs, an Okinawa‐Ishigaki pair, and an Atimonan‐Guiuan pair had relatedness estimates with an upper 95% confidence limit at or above that expected from first cousins. The related pairs found within Ishigaki and Okinawa contributed to an increase in average relatedness measured among individuals within sites in the Ryukyu Islands, with the largest average relatedness measured within Okinawa (Figure 5). Mean coefficients of relatedness for all estimators are reported in Table S3 in Supporting Information.

## 4 DISCUSSION

### 4.1 Genetic differentiation within the Kuroshio Current

Examination of genetic differentiation across the sampled sites indicates that peripheral populations in *Caesio cuning* are disjunct, despite the potential for connectivity with central sites via the Kuroshio Current. Across all sites, both the PCA and STRUCTURE analysis show strong support for at least two genetic clusters, one in Okinawa and the other containing the individuals from Ishigaki and the Philippines. A distinct genetic lineage in the Ryukyu Islands is likely sustained in part by divergent selection in marginal environmental conditions at higher latitudes. The largest portion of variance in allele frequencies (PC 1) in loci under selection separated the individuals sampled from Ishigaki and Okinawa from those sampled from the east coast of the Philippines. Putative proximity to coding regions of many of these outlier loci may indicate that conditions in peripheral populations are driving local adaptation, but megablast search results are not conclusive enough to make direct connections to functional genomic regions.

The GENELAND results supporting three clusters are likely a result of both the scale of geographic distance between sampled sites and deviations from model assumptions. When coordinate uncertainty was changed to 10 km or even 50 km, GENELAND never assigned individuals within sites to different clusters despite both the PCA and STRUCTURE results indicating that an individual sampled from Okinawa was most closely aligned to the genetic signature of Ishigaki. These results suggest a spatially explicit method of clustering is likely to be most powerful when samples are more evenly distributed across the landscape. In addition, the presence of ghost populations in both our first (unknown K) and second (fixed K) MCMC outputs could be an indication of deviations from assumptions underlying the correlated frequency model. The GENELAND authors note that ghost populations in simulated data are extremely rare and suggest the presence of them can be a clue that data depart from model assumptions (Guillot et al., 2009). A departure such as isolation‐by‐distance, which partial mantel tests indicated is present across the five sampled sites, could result in overestimating the number of genetic clusters.

The presence of isolation‐by‐distance between sites supports evidence from observations of larval behavior that *C. cuning* do not regularly disperse long distances. A previous study using mitochondrial DNA found no evidence for IBD though long‐distance dispersal was hypothesized to be rare from the low levels of mixing observed between divergent mitochondrial clades (Ackiss et al., 2013). This is the first time IBD has been documented in this species and is likely due to the resolution provided by a large SNP dataset.

Though sites in the Philippines belong to the same genetic lineage, pairwise genetic distances were significantly different between all but the two northern sites, Santa Ana and Atimonan, which may reflect a combination of geographic and oceanographic barriers to connectivity with Guiuan. Continual shoreline connects Santa Ana and Atimonan, but the ~17 km San Bernardino Strait divides the coastline between Atimonan and Guiuan. While there are likely equivalent lengths within the coastline that do not have appropriate reef habitat to support *C. cuning,* the presence of a consistent westward flow through the San Bernardino Strait (Han, Moore, Di Lorenzo, Gordon, & Lin, 2008) could prevent larval passage across the strait. A more likely driver of genetic differentiation is the presence of the NEC bifurcation south of Atimonan. The NEC bifurcation occurs between the latitudes of 11°–16.5°N (Qiu & Lukas, 1996), placing Guiuan within the southward flowing Mindanao Current. The bifurcation of the NEC has been hypothesized to restrict genetic connectivity of reef species along the east coast of the Philippines, and genetic structure supporting this has been found in a giant clam species (*Tridacna crocea*) (Ravago‐Gotanco, Magsino, & Juinio‐Meñez, 2007).

### 4.2 Edge effects in peripheral populations

While cluster analyses clearly differentiate Okinawa from the other sites, the effects of peripheral isolation were noticeable in estimates of both relatedness and effective population size in all three of the most northern sites. Only a single pair of samples from the Philippines was related at approximately the first cousin level, and that pair was sampled from two separate sites. No within‐site related individuals were sampled until Ishigaki with a marked increase of within‐site relatedness in Okinawa. In addition, finite estimates of *N*_e_ were only calculated for the three most northern sites with the smallest *N*_e_ calculated in Okinawa. Comparatively, the two most southern sites, Guiuan and Atimonan, only produced lower bound estimates for *N*_e_.

The estimates of contemporary effective population size presented here should be viewed as spatially informative rather than as accurate estimates of true effective population size in these locations. Simulations have shown precise estimation of *N*_e_ using the LD *N*_e_ method can be achieved with small populations (*N*_e_ < 200), but reliable estimates of *N*_e_ from large populations are difficult (Waples & Do, 2010). The researchers point out, however, that even the ability to estimate a lower bound for *N*_e_ can provide useful information, and in the case of the five sites sampled along the Kuroshio Current, indicates a successively decreasing number of effective breeders contributing to local populations from the center to the periphery of the species range. This pattern holds true even when sample sites are combined based on the significance of *F*_ST_ values or PCA, STRUCTURE, and GENELAND results.

Even with a trend of decreasing *N*_e_, there is no evidence that peripheral populations of *C. cuning* are currently experiencing detrimental genetic depression. No within‐ or between‐site relatedness was measured at a level greater than first cousin, and *G*_IS_ in all sites is close to zero so there is no indication of inbreeding within any of the sampled populations. However, large declines in fusilier abundance due to artisanal fishing have been previously documented in the Philippines (Alcala & Russ, 1990). If overharvest of this species were to occur in the periphery, the increased genetic differentiation and edge effects puts these populations at risk of a bottleneck event and slower recovery times.

Only a handful of studies have examined genetic connectivity of tropical organisms in the vicinity of the Kuroshio Current, with results indicating varying genetic patterns. One analysis of a species of seagrass (*Syringodium isoetifolium*) indicates that it exhibits very similar central‐peripheral patterns to *C. cuning* (Kurokochi et al., 2016). A few support the Kuroshio Current as a homogenizing force across continuous populations in the Philippines and the Ryukyu Islands. Though not explicitly examining population dynamics across this central‐peripheral junction, a microsatellite analysis of a seastar (*Acanthaster planci*) showed no differentiation between a site in the central Philippines and the Ryukyu Islands and no decrease in genetic diversity (Yasuda et al.., 2009). Microsatellite analyses of three species of seagrass (*Cymodocea serrulata, Cymodocea rotunda,* and *Enhalus acoroides*) indicate that recruitment to the Ryukyus occurs from the northeastern Philippines (Arriesgado et al., 2015, 2016 ; Nakajima et al., 2014). Reduced genetic diversity was only found in Ryukyu Island populations of *E. acoroides* and *C. serrulata*, however, providing further evidence that many tropical taxa may not experience measurable edge effects in peripheral populations within the Kuroshio Current.

The results of this study indicate that genetic patterns from central to peripheral populations of *C. cuning* within the Kuroshio Current are influenced by both dispersal ability and local adaptation in marginal habitat. With the predominance of this western‐boundary current and the shelf topography in this region, no known physical barrier has limited pelagic larval dispersal, even during the low sea levels of the Pleistocene epoch (Voris, 2000). Given the dominance of a single lineage in the Philippines, continuous habitat appears to be influential enough to prevent large amounts of genetic divergence. Once this continuous shoreline habitat gives way to disjunct islands, patterns of isolation‐by‐distance, and peripheral divergence arise. Enough dispersal from the Philippines to Ishigaki appears to be occurring to make individuals in this island more similar to those in the Philippines than Okinawa, even though Okinawa is nearly 200 km closer to Ishigaki. However, what roles successively decreasing effective population sizes across these disjunct stepping‐stones and localized selective pressures in increasingly subtropical habitat play in shaping this pattern cannot be determined from these data. Studies of additional taxa with a range of dispersal potential and peripheral genetic diversity or comparative studies examining differences in gene expression in individuals in central and peripheral populations will be needed to further untangle the relative influences of dispersal ability and selective pressure in shaping patterns in peripheral populations of tropical species in the Kuroshio Current.

## CONFLICT OF INTEREST

None declared.

## AUTHOR CONTRIBUTIONS

A.S.A. conceived this study; A.S.A., Y.A., K.T., and K.E.C. collected samples; A.S.A. performed library preparation, genetic analyses, and analyzed data; A.S.A, C.E.B, and K.E.C. wrote the manuscript with contributions from Y.A. and M.D.S.; all the authors approved the final manuscript.

## DATA ACCESSIBILITY

Raw sequence data in fastq format and corresponding metadata have been archived with the publically accessible Genomic Observatories Metadatabase (GeOMe, GUID: https://n2t.net/ark:/21547/BdX2, NCBI Project ID: PRJNA497108).
