# Supplementary material for: Genetic patterns in peripheral marine populations of the fusilier fish Caesio cuning within the Kuroshio Current
Source: Ecol Evol. 2018 Nov 14;8(23):11875–86. doi: 10.1002/ece3.4644 (PMC6303744; doi:10.1002/ece3.4644)
Supplement: Supplementary file 1 [file ECE3-8-11875-s001.docx]

**Genetic patterns in peripheral marine populations of the fusilier fish *Caesio cuning* within the Kuroshio Current**

Amanda S. Ackiss, Christopher E. Bird, Yuichi Akita, Mudjekeewis D. Santos, Katsunori Tachihara, Kent E. Carpenter

| **Sample pair** | ***H*_O_** | **Estimators** | **r** | **95% CI** |
| --- | --- | --- | --- | --- |
|  |  |  |  |  |
| San_007- | 0.272 | Ritland (1996) | 0.934 | 0.865-1.006 |
| San_008 | 0.262 | Milligan (2003)† | 0.967 | 0.948-0.987 |
|  |  | Wang (2007)‡ | 0.967 | 0.948-0.991 |
|  |  |  |  |  |

**Supplementary Tables**

**Table S1.** Coefficients of relatedness (r) from the r package ‘related’ for San_007 and San_008. Estimates of relatedness are consistent with the individual San_007 being sampled twice.

† dyadic likelihood estimator

‡ triadic likelihood estimator

| **Query ID** | **Hit ID** | **Hit Accession #** | **% Identity** | **Alignment**  **Length** | **Mismatches** | **Gap opens** | **Query start** | **Query end** | **Hit Start** | **Hit end** | **e-value** | **bit score** |
| --- | --- | --- | --- | --- | --- | --- | --- | --- | --- | --- | --- | --- |
| dDocent_Contig_11633_F | gi\|374428415\|emb\|FQ310507.3\| | FQ310507.3 | 93.827 | 81 | 3 | 2 | 1 | 81 | 2843967 | 2844045 | 2.97E-24 | 121 |
| dDocent_Contig_20147_F | gi\|397776255\|gb\|JQ780820.1\| | JQ780820.1 | 95.918 | 49 | 1 | 1 | 12 | 59 | 2003 | 1955 | 1.81E-11 | 78.7 |
| dDocent_Contig_20147_F | gi\|374428414\|emb\|FQ310506.3\| | FQ310506.3 | 95.918 | 49 | 1 | 1 | 12 | 59 | 1588073 | 1588121 | 1.81E-11 | 78.7 |
| dDocent_Contig_20147_F | gi\|374428414\|emb\|FQ310506.3\| | FQ310506.3 | 93.878 | 49 | 2 | 1 | 12 | 59 | 11848600 | 11848552 | 8.44E-10 | 73.1 |
| dDocent_Contig_20147_F | gi\|374428414\|emb\|FQ310506.3\| | FQ310506.3 | 83.117 | 77 | 7 | 6 | 12 | 86 | 11175136 | 11175064 | 1.41E-07 | 65.8 |
| dDocent_Contig_20147_F | gi\|374428414\|emb\|FQ310506.3\| | FQ310506.3 | 83.117 | 77 | 7 | 6 | 12 | 86 | 11893116 | 11893044 | 1.41E-07 | 65.8 |
| dDocent_Contig_20147_F | gi\|374428414\|emb\|FQ310506.3\| | FQ310506.3 | 97.222 | 36 | 1 | 0 | 23 | 58 | 12070153 | 12070188 | 1.83E-06 | 62.1 |
| dDocent_Contig_20147_F | gi\|374428414\|emb\|FQ310506.3\| | FQ310506.3 | 97.222 | 36 | 1 | 0 | 24 | 59 | 12571976 | 12572011 | 1.83E-06 | 62.1 |
| dDocent_Contig_20147_F | gi\|712042627\|gb\|KJ546039.1\| | KJ546039.1 | 93.878 | 49 | 2 | 1 | 12 | 59 | 1609 | 1657 | 8.44E-10 | 73.1 |
| dDocent_Contig_20147_F | gi\|1104859873\|dbj\|LC056058.1\| | LC056058.1 | 95.556 | 45 | 1 | 1 | 16 | 59 | 28686 | 28642 | 3.04E-09 | 71.3 |
| dDocent_Contig_20147_F | gi\|1065196124\|gb\|KU236380.1\| | KU236380.1 | 100 | 37 | 0 | 0 | 23 | 59 | 919 | 883 | 1.09E-08 | 69.4 |
| dDocent_Contig_20147_F | gi\|657596110\|ref\|XM_008304520.1\| | XM_008304520.1 | 95.349 | 43 | 2 | 0 | 17 | 59 | 3836 | 3878 | 1.09E-08 | 69.4 |
| dDocent_Contig_20147_F | gi\|429508162\|gb\|JQ710660.1\| | JQ710660.1 | 100 | 37 | 0 | 0 | 23 | 59 | 84 | 48 | 1.09E-08 | 69.4 |
| dDocent_Contig_20147_F | gi\|1108984874\|ref\|XM_019270578.1\| | XM_019270578.1 | 97.222 | 36 | 1 | 0 | 23 | 58 | 1051 | 1086 | 1.83E-06 | 62.1 |
| dDocent_Contig_20147_R | gi\|374428414\|emb\|FQ310506.3\| | FQ310506.3 | 88.889 | 63 | 7 | 0 | 18 | 80 | 10013362 | 10013300 | 1.81E-11 | 78.7 |
| dDocent_Contig_34240_R | gi\|1108997576\|ref\|XM_010731103.2\| | XM_010731103.2 | 94.505 | 91 | 1 | 3 | 1 | 91 | 7863 | 7777 | 2.95E-29 | 137 |
| dDocent_Contig_34240_R | gi\|657541357\|ref\|XM_008278362.1\| | XM_008278362.1 | 96.154 | 78 | 1 | 2 | 14 | 91 | 8443 | 8368 | 6.39E-26 | 126 |
| dDocent_Contig_41922_R | gi\|992195141\|ref\|XM_015604406.1\| | XM_015604406.1 | 90.698 | 86 | 8 | 0 | 2 | 87 | 1507 | 1592 | 1.38E-22 | 115 |
| dDocent_Contig_7771_R | gi\|374428414\|emb\|FQ310506.3\| | FQ310506.3 | 94.34 | 53 | 2 | 1 | 1 | 52 | 10385797 | 10385849 | 5.05E-12 | 80.5 |
| dDocent_Contig_7771_R | gi\|374428414\|emb\|FQ310506.3\| | FQ310506.3 | 89.286 | 56 | 5 | 1 | 1 | 55 | 8161016 | 8160961 | 1.09E-08 | 69.4 |
| dDocent_Contig_7771_R | gi\|374428416\|emb\|FQ310508.3\| | FQ310508.3 | 91.071 | 56 | 4 | 1 | 1 | 55 | 13849368 | 13849313 | 2.35E-10 | 75 |
| dDocent_Contig_7771_R | gi\|374428415\|emb\|FQ310507.3\| | FQ310507.3 | 87.5 | 56 | 6 | 1 | 1 | 55 | 8852433 | 8852488 | 5.08E-07 | 63.9 |

**Table S2**. BLASTN alignment summary for RAD tags associated with 5 of 36 outlier loci.

**Table S3**. Mean coefficients of relatedness (r) from the r package ‘related’ for individuals within populations.

| **Location** | **r** | | | | |
| --- | --- | --- | --- | --- | --- |
|  | **Ritland (1996)** | **Milligan (2003)**† | | **Wang(2007)**‡ | |
|  |  |  | |  | |
| OKI | 0.0241 | 0.0322 | | 0.0317 | |
| ISH | 0.0023 | 0.0155 | | 0.0152 | |
| SAN | -0.0041 | 0.0114 | | 0.0111 | |
| ATI | -0.0018 | 0.0126 | | 0.0122 | |
| GUI | -0.0006 | 0.0134 | | 0.0131 | |
|  |  | |  | |  |

† dyadic likelihood estimator

‡ triadic likelihood estimator
