# Supplementary material for: Genetic patterns in peripheral marine populations of the fusilier fish Caesio cuning within the Kuroshio Current
Source: Ecol Evol. 2018 Nov 14;8(23):11875–86. doi: 10.1002/ece3.4644 (PMC6303744; doi:10.1002/ece3.4644)
Supplement: Supplementary file 2 [file ECE3-8-11875-s002.docx]

# This script has been modified for NeEstimator v2.1 from the original script

# available as supplementary material from Candy et al. 2015.

import sys

import re

def dirty_to_clean_r2_distr(r2_dirty_file):

with open(r2_dirty_file, "r") as dirty_R2:

clean_r2_filename="clean_{}".format(r2_dirty_file)

clean_r2_file=open(clean_r2_filename, "w")

title_string="locus1\tlocus2\tsamp_size\tMean_r2\tr2_drift\n"

clean_r2_file.write(title_string)

for line in dirty_R2:

line = line.strip()

if re.match(r'[0-9]+\s',line): #starts with num

split_r2_line=line.split()

locus1=split_r2_line[0]

locus2=split_r2_line[1]

samp_size=split_r2_line[4]

mean_r2=split_r2_line[5]

r2_drift=split_r2_line[6]

combo_line=locus1+"\t"+locus2+"\t"+samp_size+"\t"+mean_r2+"\t"+r2_drift+"\n"

clean_r2_file.write(combo_line)

clean_r2_file.close()

filename=sys.argv[1]

dirty_to_clean_r2_distr(filename)

print(filename)
